# Treatment decisions, not patient demographics, predict a positive pregnancy test after autologous IVF: a retrospective cohort study from northern India

**DOI:** 10.3389/fpubh.2026.1922605

**Published:** 2026-10-05

**Authors:** Rajesh Kamath, Gwendolen Rodrigues, Saurabh Singh, Nahima Akthar, Rakshitha R. Shenoy, Sandesh Kini

**Affiliations:** 1 Department of Healthcare and Hospital Management, Prasanna School of Public Health, Manipal Academy of Higher Education, Manipal, India; 2 Directorate of Online Education, Manipal Academy of Higher Education, Manipal, India; 3 Department of Community Medicine, Kasturba Medical College, Manipal Academy of Higher Education, Manipal, India; 4 Department of Pediatrics, Kasturba Medical College, Manipal Academy of Higher Education, Manipal, India

**Keywords:** assisted reproductive technology, clinical pregnancy, frozen embryo transfer, India, intracytoplasmic sperm injection, in-vitro fertilisation

## Abstract

**Background:**

Infertility affects about one in six people worldwide over a lifetime, and *in vitro* fertilisation (IVF) is the mainstay of treatment for many of them. In India most IVF is delivered in private clinics and paid for out of pocket. The question is which factors determine whether an IVF cycle produces a pregnancy: the characteristics a patient arrives with, or the treatment decisions made once she is there.

**Methods:**

We conducted a retrospective cohort study of 288 women who underwent IVF at a private fertility centre in Faridabad, Haryana, between April 2023 and October 2024. One record was analysed per woman, taken from her most recent cycle. The outcome was a positive serum beta-human chorionic gonadotropin (beta-hCG) test, the last outcome routinely documented at the centre. Donor-oocyte (recipient) cycles were analysed separately from autologous cycles, because success in a donor cycle depends on the donor rather than on the patient. Categorical associations were tested using Fisher’s exact test. Independent predictors were identified using multivariable binomial logistic regression.

**Results:**

Seventy-seven cycles (26.7%) used donor oocytes and 211 (73.3%) were autologous. The positive beta-hCG rate was 70.6% in autologous cycles and 54.5% in donor cycles (*p* = 0.016). Within autologous cycles, two treatment decisions were associated with the outcome: intracytoplasmic sperm injection (ICSI) versus conventional IVF (76.0% versus 46.2%, *p* < 0.001) and frozen versus fresh embryo transfer (74.9% versus 51.3%, *p* = 0.006). No demographic characteristic was associated with the outcome. After adjustment (*N* = 210), conventional IVF carried lower odds than ICSI (adjusted odds ratio 0.16, 95% CI 0.06–0.42, *p* < 0.001) and frozen transfer higher odds than fresh (adjusted odds ratio 2.80, 95% CI 1.30–6.04, *p* = 0.009). Oocyte yield was marginally associated (adjusted odds ratio 1.11 per oocyte, 95% CI 1.00–1.22, *p* = 0.041). In absolute terms, ICSI corresponded to 32.5 percentage points and frozen transfer to 18.5 percentage points. Age was not associated with the outcome, although autologous cycles spanned only 22–42 years and the study was underpowered to detect age effects.

**Conclusion:**

In autologous cycles at this centre, treatment decisions rather than patient demographics predicted a positive pregnancy test. The cohort was socioeconomically narrow and drawn from a single private centre. The absence of demographic effects should therefore not be read as evidence that social position does not matter. Social position might operate earlier, in determining who reaches a fertility centre at all.

## 1 Introduction

Infertility affects about one in six people worldwide at some point in their lives (1). In India the burden is substantial and the response is uneven (2, 3). Community-based estimates put primary infertility in urban central India at a level that implies a large unmet need (4), and assisted reproduction now contributes measurably to births worldwide (5). Assisted reproductive technology has expanded quickly, but almost entirely through private providers, and almost entirely on an out-of-pocket basis (6, 7). A cycle can cost more than many Indian households earn in a year. Public insurance does not cover it.

That financing structure shapes who is treated. It might also shape what is known. Most Indian ART data come from private centres serving patients who could pay, which makes it difficult to separate the effect of a patient’s characteristics from the effect of the treatment she receives. Two treatment decisions dominate contemporary practice. The first is whether to fertilise by intracytoplasmic sperm injection (ICSI) or by conventional IVF. ICSI was developed for male-factor infertility. Its use seems to have expanded well beyond that indication. Randomised and comparative evidence in non-male-factor couples has not shown it to be superior (8–10). The second is whether to transfer fresh or frozen embryos. Frozen transfer has grown rapidly on the argument that a non-stimulated endometrium is more receptive (11).

Alongside these sit the patient’s own characteristics: age, body mass index, socioeconomic position, education, occupation, and the type and duration of infertility. Age in particular is among the best-established determinants of IVF success (12). This study asked which of these predicted a positive pregnancy test in a private fertility centre in northern India. Because donor-oocyte cycles break the link between the patient’s age and the quality of the oocyte, they were analysed separately throughout.

## 2 Materials and methods

### 2.1 Study design and setting

This was a retrospective cohort study conducted at a private fertility centre in Faridabad, Haryana, in northern India. The centre serves a predominantly urban, self-paying population. Clinical records covering cycles performed between April 2023 and October 2024 were reviewed. Records were abstracted in January 2025 onto a structured proforma and anonymised before analysis.

### 2.2 Participants

Women aged 21–50 years who had completed at least one IVF cycle at the centre during the study period were eligible. Women who attended only for initial consultation without proceeding to IVF were excluded. The centre operates internal completeness checks before a record is filed, and no record was excluded for incompleteness. One record was analysed per woman. Where a woman had undergone more than one cycle, the most recent cycle was taken. The unit of analysis is therefore the woman, not the cycle, and the results should be read as the outcome of a woman’s latest attempt rather than as a per-cycle success rate.

### 2.3 Outcome definition

The outcome was a positive serum beta-hCG test following embryo transfer, recorded as positive or negative. This is a biochemical pregnancy. It is not clinical pregnancy, ongoing pregnancy or live birth, none of which could be ascertained. The centre does not hold an ultrasound licence; imaging is performed at partner facilities and is not captured in the clinic record, so no gestational-age confirmation exists in the dataset. Beta-hCG was the last outcome documented for the cohort as a whole. No cycle was recorded as negative on the basis of non-attendance; all outcomes reflect a documented test result. There was no follow-up beyond this test. Each record contributes a single outcome measured after embryo transfer, and no cycle was followed to ultrasound, to delivery, or to any later endpoint.

### 2.4 Donor-oocyte cycles

Cycles in which the patient received donated oocytes are recorded at the centre under a recipient designation, supported by separate donor documentation. Most recipients do not undergo ovarian stimulation or oocyte retrieval, and the recorded oocyte yield is zero. In six cycles the patient underwent retrieval first; embryo quality was judged inadequate, and the patient was offered and accepted donated oocytes. The recorded yield in those six records refers to that abandoned autologous attempt. The transfer used donated oocytes in all 77 cycles. Because the probability of fertilisation and implantation in such a cycle reflects the donor’s age and ovarian reserve rather than the patient’s, pooling donor and autologous cycles would confound any analysis of patient characteristics. Autologous cycles therefore form the primary analysis, and donor cycles are reported separately.

### 2.5 Variables

Demographic variables were age, body mass index, education, occupation, socioeconomic status and type of infertility. Body mass index was classified using Asian-specific cut-offs. All five categories are reported descriptively in Table 1. For modelling it was dichotomised as normal (18.5–22.9 kg/m^2^) or abnormal, because the underweight (*n* = 4) and obese II (*n* = 21) cells were too small to support stable category-specific estimates. Socioeconomic status was classified using the modified Kuppuswamy scale as updated for 2024 (13). The scale combines the education and the occupation of the head of the family with total monthly family income. The income component was indexed to the Consumer Price Index for Industrial Workers for March 2024 (183.6), and education and occupation were scored by the standard classification. Income was taken from the financial counselling form held in the patient file, a routine record in a self-paying setting. The education and occupation reported separately in Tables 1,2 and 3 are those of the patient herself, not of the head of the family, and are therefore distinct from the components of the socioeconomic score. Collinearity between them was assessed directly (Section 2.6). Clinical and treatment variables were duration of infertility, cause of infertility, number of previous pregnancies, ovarian stimulation protocol, number of oocytes retrieved, number of embryos transferred, fertilisation method (ICSI or conventional IVF), embryo transfer type (fresh or frozen) and cycle number.

**Table 1:** Characteristics of the autologous cohort (*n* = 211).

Characteristic	Category	N	%
Socioeconomic status	Upper	52	24.6
	Upper middle	100	47.4
	Lower middle	34	16.1
	Lower	25	11.8
Occupation	Homemaker	114	54.0
	Employed/other	97	46.0
Education	No schooling	17	8.1
	Middle school	36	17.1
	High school	36	17.1
	Graduation	98	46.4
	Post graduation	24	11.4
Type of infertility	Primary	145	68.7
	Secondary	66	31.3
Previous pregnancies	0	158	74.9
	1	41	19.4
	2	12	5.7
Ovulation stimulation	Long protocol–FSH	146	69.2
	Antagonist protocol–HMG	65	30.8
Embryos transferred	1	10	4.7
	2	188	89.1
	3	13	6.2
Fertilisation method	ICSI	172	81.5
	Conventional IVF	39	18.5
Embryo transfer type	Fresh	39	18.5
	Frozen	171	81.0
BMI (Asian cut-offs)	Underweight	4	1.9
	Normal	59	28.0
	Overweight	60	28.4
	Obese I	67	31.8
	Obese II	21	10.0
Treatment cycle number	1	153	72.5
	2	40	19.0
	3	17	8.1
	4	1	0.5
Outcome	Positive beta-hCG	149	70.6
	Negative	62	29.4

Socioeconomic status classified using the modified Kuppuswamy scale (2024 update; income component indexed to CPI-IW March 2024 = 183.6), with income taken from the financial counselling form in the patient file. BMI classified using Asian-specific cut-offs (normal = 18.5–22.9 kg/m^2^). One cycle used a mixed fresh-and-frozen transfer and is not shown under embryo transfer type. The analytic sample for models involving transfer type is 210.

**Table 2:** Univariate associations with a positive beta-hCG, autologous cycles (*N* = 210).

Characteristic	Category	Positive/total (%)	*p*-value
Age group (years)	21–25	20/30 (66.7)	0.889
	26–30	44/64 (68.8)	
	31–35	58/81 (71.6)	
	36–42	26/35 (74.3)	
Socioeconomic status	Upper	41/51 (80.4)	0.198
	Upper middle	64/100 (64.0)	
	Lower middle	24/34 (70.6)	
	Lower	19/25 (76.0)	
Occupation	Homemaker	78/113 (69.0)	0.651
	Employed/other	70/97 (72.2)	
Education	School or less	58/88 (65.9)	0.175
	Graduate	75/98 (76.5)	
	Postgraduate	15/24 (62.5)	
Type of infertility	Primary	100/144 (69.4)	0.745
	Secondary	48/66 (72.7)	
Previous pregnancies	0	108/157 (68.8)	0.255
	1	29/41 (70.7)	
	2	11/12 (91.7)	
BMI	Normal	43/59 (72.9)	0.737
	Abnormal	105/151 (69.5)	
Stimulation protocol	Long protocol–FSH	106/145 (73.1)	0.252
	Antagonist protocol–HMG	42/65 (64.6)	
Embryos transferred	1	8/10 (80.0)	0.134
	2	134/187 (71.7)	
	3	6/13 (46.2)	
Fertilisation method	ICSI	130/171 (76.0)	<0.001
	Conventional IVF	18/39 (46.2)	
Embryo transfer type	Fresh	20/39 (51.3)	0.006
	Frozen	128/171 (74.9)	

*p*-values from Fisher’s exact test, with the Freeman–Halton extension for tables larger than 2 × 2. Unadjusted for multiple comparisons; to be read as exploratory.

**Table 3:** Fully adjusted multivariable logistic regression including all demographic characteristics, autologous cycles (*N* = 210).

Predictor	Adjusted OR (95% CI)	*p*-value
Conventional IVF (vs. ICSI)	0.16 (0.06, 0.45)	<0.001
Frozen transfer (vs. fresh)	2.61 (1.17, 5.82)	0.019
Oocytes retrieved (per oocyte)	1.11 (1.00, 1.22)	0.047
Duration of infertility (per year)	0.92 (0.83, 1.03)	0.131
Age (per year)	1.03 (0.94, 1.13)	0.563
Abnormal BMI (vs. normal)	0.96 (0.45, 2.06)	0.912
Upper SES (vs. upper middle)	1.35 (0.53, 3.48)	0.528
Lower-middle SES (vs. upper middle)	0.86 (0.31, 2.37)	0.768
Lower SES (vs. upper middle)	1.12 (0.35, 3.58)	0.846
Employed/other (vs. homemaker)	1.11 (0.55, 2.26)	0.772
Secondary infertility (vs. primary)	1.20 (0.54, 2.66)	0.661
Antagonist protocol–HMG (vs. long protocol–FSH)	0.63 (0.30, 1.31)	0.214

SES, socioeconomic status; BMI, body mass index; OR, odds ratio; CI, confidence interval. Events per variable 12.3.

### 2.6 Statistical analysis

Continuous variables are summarised as mean with standard deviation and as median with interquartile range. Categorical variables are summarised as counts and percentages. Categorical associations with a positive beta-hCG were tested using Fisher’s exact test, with the Freeman–Halton extension for tables larger than two by two. Independent predictors were identified using multivariable binomial logistic regression. The primary model was restricted to autologous cycles and included fertilisation method, embryo transfer type, age, oocyte yield and duration of infertility. A second model added body mass index, socioeconomic status, occupation, type of infertility and stimulation protocol. Odds ratios are reported with 95% confidence intervals; average marginal effects are reported as absolute risk differences to aid clinical interpretation. Model fit was assessed using the McFadden and Nagelkerke pseudo-*R*^2^ statistics. Multicollinearity was assessed using variance inflation factors, summarised by their mean and maximum. The events-per-variable ratio is reported for each model. Analyses were conducted in R version 4.4.1 and jamovi version 2.6.23; a significance threshold of *p* < 0.05 was used, and *p*-values from univariate screening are presented without adjustment for multiple comparisons and should be read as exploratory. Two sensitivity analyses were performed. The primary model was repeated after excluding male-factor cases, in which ICSI is mandatory, and after adding cycle number, coded as first versus second or later.

### 2.7 Sample size

A minimum of 288 records was estimated for a single-proportion calculation at 95% confidence and 5% absolute precision. The realised sample was also constrained by the number of complete records available within the study period. The observed proportion (70.6% in autologous cycles) differs from the assumed proportion, so the events-per-variable ratio is reported as the more relevant indicator of adequacy for the regression models.

### 2.8 Ethics

The study was approved by the Institutional Ethics Committee of Kasturba Medical College and Kasturba Hospital, Manipal (IEC2: 629/2024), on 5 December 2024, as a retrospective review of existing clinical records. Permission was obtained from the medical director of the fertility centre. No patient was contacted at any stage. Each record was assigned a unique anonymised identifier before analysis, and individual informed consent was not obtained.

## 3 Results

### 3.1 Cohort composition

Of 288 women, 77 (26.7%) underwent donor-oocyte cycles and 211 (73.3%) underwent autologous cycles. The two groups differed in age. Autologous patients had a mean age of 30.8 years (SD 4.6, range 22–42). Donor recipients had a mean age of 39.0 years (SD 5.2, range 24–49). Every woman in the cohort older than 42 years received donor oocytes. The positive beta-hCG rate was 70.6% (149/211) in autologous cycles and 54.5% (42/77) in donor cycles (*p* = 0.016). All subsequent analyses are restricted to autologous cycles unless stated otherwise. One autologous record used a mixed fresh-and-frozen transfer and was excluded from models involving transfer type, giving an analytic sample of 210. Characteristics of the autologous cohort are shown in Table 1 and Table 4. Most women were in the upper or upper-middle socioeconomic strata (72.0%). Primary infertility accounted for 68.7% of cases. ICSI was used in 81.5% of cycles and frozen transfer in 81.0%. Two embryos were transferred in 89.1% of cycles; the centre did not operate an elective single-embryo-transfer policy during the study period.

**Table 4:** Continuous variables, autologous cohort (*n* = 211).

Variable	Mean (SD)	Median (IQR)	Range
Age (years)	30.78 (4.63)	31.0 (28.0–34.0)	22–42
Duration of infertility (years)	6.98 (3.57)	7.0 (4.0–9.5)	1–18
Oocytes retrieved	8.36 (3.93)	9.0 (5.0–11.0)	1–18

### 3.2 Univariate associations

Two variables were associated with a positive beta-hCG, and both were treatment decisions (Table 2). ICSI achieved a higher rate than conventional IVF: 76.0% versus 46.2% (*p* < 0.001). Frozen transfer achieved a higher rate than fresh transfer: 74.9% versus 51.3% (*p* = 0.006).

No demographic or clinical characteristic reached significance. Age group (*p* = 0.889), socioeconomic status (*p* = 0.198), occupation (*p* = 0.651), education (*p* = 0.175), type of infertility (*p* = 0.745), number of previous pregnancies (*p* = 0.255), stimulation protocol (*p* = 0.252), number of embryos transferred (*p* = 0.134) and body mass index (*p* = 0.737) were all non-significant. Rates across age groups ranged from 66.7% in the 21–25 year group to 74.3% in the 36–42 year group (Figure 1A), with no downward gradient. This should not be read as evidence that age does not matter. Autologous cycles spanned only 22–42 years, only 35 women were aged 36 or above, and the study had limited power to detect an age effect within that narrow range.

**Figure 1:**
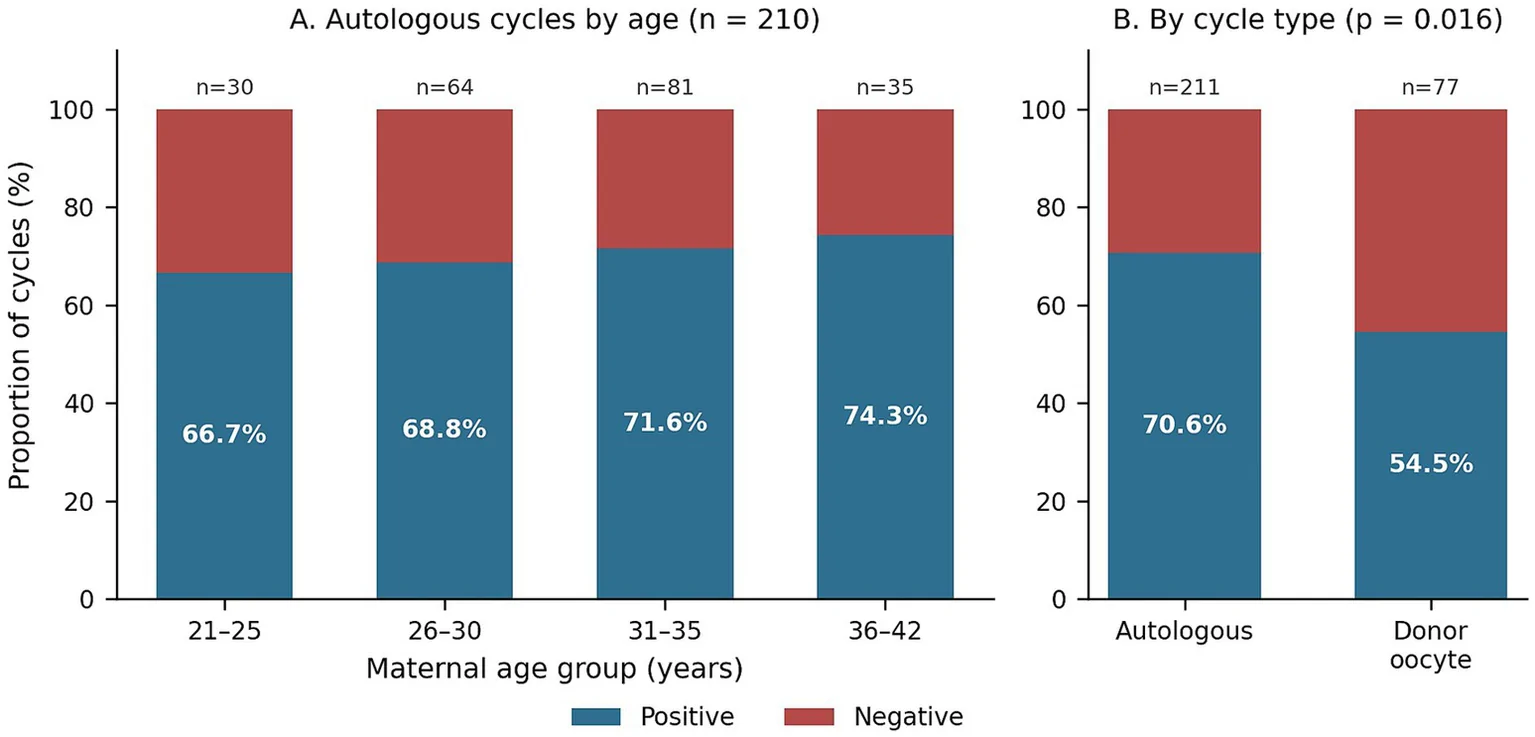
Positive beta-hCG by maternal age group in autologous cycles [panel **(A)**] and by cycle type [panel **(B)**]. Panel A shows the proportion of autologous cycles with a positive and a negative result in each age group (*n* = 210; one cycle with a mixed fresh-and-frozen transfer is excluded). Panel B compares autologous with donor-oocyte cycles (*n* = 288).

### 3.3 Independent predictors

Two factors survived adjustment (Table 5; *N* = 210). Conventional IVF carried markedly lower odds than ICSI: adjusted odds ratio 0.16 (95% CI 0.06–0.42, *p* < 0.001). Frozen transfer carried higher odds than fresh: adjusted odds ratio 2.80 (95% CI 1.30–6.04, *p* = 0.009). Oocyte yield was marginally associated (adjusted odds ratio 1.11 per additional oocyte, 95% CI 1.00–1.22, *p* = 0.041). Age (*p* = 0.488) and duration of infertility (*p* = 0.063) were not significant.

**Table 5:** Primary multivariable logistic regression, autologous cycles (outcome: positive beta-hCG; *N* = 210).

Predictor	Adjusted OR (95% CI)	Crude OR (95% CI)	Marginal effect	*p*-value
Conventional IVF (vs. ICSI)	0.16 (0.06, 0.42)	0.27 (0.13, 0.56)	−32.5 pp	<0.001
Frozen transfer (vs. fresh)	2.80 (1.30, 6.04)	2.83 (1.38, 5.79)	+18.5 pp	0.009
Oocytes retrieved (per oocyte)	1.11 (1.00, 1.22)	1.02 (0.94, 1.10)	+1.8 pp	0.041
Age (per year)	1.03 (0.95, 1.12)	1.02 (0.96, 1.09)	+0.5 pp	0.488
Duration of infertility (per year)	0.91 (0.82, 1.01)	0.95 (0.87, 1.03)	−1.7 pp	0.063

Reference categories: ICSI (fertilisation method) and fresh (embryo transfer type). Marginal effect is the average marginal effect, expressed as an absolute difference in percentage points (pp). Events per variable 29.6. OR, odds ratio; CI, confidence interval.

In absolute terms, ICSI corresponded to a difference of 32.5 percentage points in the probability of a positive test (*p* < 0.001) and frozen transfer to 18.5 percentage points (*p* = 0.005). Each additional oocyte retrieved corresponded to 1.8 percentage points (*p* = 0.035).

Adding all demographic characteristics left both treatment effects essentially unchanged and added no significant predictor (Table 3): conventional IVF 0.16 (95% CI 0.06–0.45, *p* < 0.001) and frozen transfer 2.61 (95% CI 1.17–5.82, *p* = 0.019). Socioeconomic status, occupation, body mass index, type of infertility and stimulation protocol were all non-significant.

### 3.4 Model diagnostics

Multicollinearity was not a problem. Variance inflation factors were low in the primary model (mean 1.3, maximum 1.5) and in the fully adjusted model (mean 1.3, maximum 1.7). Because socioeconomic status is a composite measure that could nonetheless overlap with the patient’s own education and occupation, the fully adjusted model was refitted with the patient’s education entered alongside her occupation and socioeconomic status; variance inflation factors remained low (mean 1.7, maximum 3.0), well below the conventional threshold of 10. The primary model had a residual deviance of 226.3 and an Akaike information criterion of 238.3. McFadden’s pseudo-R^2^ was 0.112 and Nagelkerke’s was 0.181. With 148 events and five predictors, the events-per-variable ratio was 29.6 for the primary model and 12.3 for the fully adjusted model, both above the conventional minimum of 10.

### 3.5 Sensitivity analyses

ICSI is mandatory in male-factor infertility, so its apparent advantage could be an artefact of indication. The model was repeated after excluding the 48 autologous male-factor cases, leaving 162 cycles in which ICSI was a discretionary choice. The advantage held (Table 6): adjusted odds ratio 0.17 for conventional IVF (95% CI 0.06–0.50, *p* = 0.001). The frozen-transfer effect strengthened: adjusted odds ratio 3.79 (95% CI 1.63–8.82, *p* = 0.002). Cycle number did not account for the treatment effects. Adding it to the primary model left conventional IVF at 0.16 (95% CI 0.06–0.41, *p* < 0.001), frozen transfer at 2.79 (95% CI 1.29–6.04, *p* = 0.009) and oocyte yield at 1.11 (95% CI 1.00–1.22, *p* = 0.041). A second or later cycle was not associated with the outcome (adjusted odds ratio 1.37, 95% CI 0.66–2.88, *p* = 0.401).

**Table 6:** Sensitivity analysis restricted to non-male-factor autologous cycles (*N* = 162).

Predictor	Adjusted OR (95% CI)	*p*-value
Conventional IVF (vs. ICSI)	0.17 (0.06, 0.50)	0.001
Frozen transfer (vs. fresh)	3.79 (1.63, 8.82)	0.002
Oocytes retrieved (per oocyte)	1.08 (0.96, 1.20)	0.190
Age (per year)	1.01 (0.91, 1.11)	0.893
Duration of infertility (per year)	0.90 (0.80, 1.01)	0.067

Excludes 48 autologous cycles with a male-factor indication (azoospermia, oligospermia, asthenoteratozoospermia or male factor), in which ICSI is mandatory. Events per variable 22.2.

### 3.6 Donor-oocyte cycles

Donor cycles are summarised in Table 7. The positive beta-hCG rate was 54.5% (42/77), lower than in autologous cycles despite the donated oocytes (Figure 1B). Recipients were older (mean 39.0 years) and received three embryos more often (40.3% versus 6.2%). Because outcomes in these cycles depend on donor characteristics that were not available for analysis, no predictive modelling was attempted.

**Table 7:** Donor-oocyte (recipient) cycles (*n* = 77).

Characteristic	Category	*n* (%)
Fertilisation method	ICSI	62 (80.5)
	Conventional IVF	15 (19.5)
Embryo transfer type	Fresh	29 (37.7)
	Frozen	48 (62.3)
Embryos transferred	1	1 (1.3)
	2	45 (58.4)
	3	31 (40.3)
Outcome	Positive beta-hCG	42 (54.5)
	Negative	35 (45.5)

Recipient mean age 39.0 years (SD 5.2, range 24–49). Recorded oocyte yield is zero in 71 cycles; in six, retrieval preceded a switch to donated oocytes after poor embryo quality. The transfer used donated oocytes in all 77 cycles. Positive beta-hCG rate versus autologous cycles: 54.5% vs 70.6%, *p* = 0.016.

## 4 Discussion

In autologous cycles at this centre, the outcome was predicted by what was done, not by who the patient was. Two treatment decisions were independently associated with a positive beta-hCG. No demographic characteristic was. ICSI was used in 81.5% of these cycles and frozen transfer in 81.0%. Both are decisions taken by the clinic, not characteristics the patient arrives with. The ICSI finding runs against much of the randomised evidence. A large trial in non-male-factor infertility found no advantage of ICSI over conventional IVF (8). A split-insemination study of sibling oocytes similarly found no difference (9). A comparative study in non-male-factor normoresponders found a higher pregnancy rate with conventional IVF (10). A registry analysis of 275,825 first cycles reported lower adjusted odds of live birth with ICSI than with conventional insemination where the indication was female-only infertility (14). A more recent multicentre trial of 1,064 couples with normal semen parameters reported live birth in 35% with ICSI and 31% with conventional IVF, a difference that was not significant (15). Registry evidence points both ways. In United States surveillance data, ICSI in cycles without male-factor infertility was associated with slightly lower live birth, 36.5% against 39.2% (16). In United Kingdom registry data covering all indications, ICSI was associated with higher odds of live birth (17). Our finding survived the most obvious challenge to it: restricting the analysis to non-male-factor cases, where ICSI was discretionary, left the effect essentially unchanged.

This might still not be a biological finding. The explanation could well be centre-specific.

Laboratory expertise with ICSI, clinician preference, or sperm parameters not recorded in the dataset might drive both the decision to use ICSI and the likelihood of fertilisation. ICSI is more expensive and more invasive than conventional IVF (18). The association seen here should not be taken as evidence that ICSI is superior in non-male-factor infertility. It seems to indicate the need for prospective studies in Indian settings. In the meantime, the established position holds: match the fertilisation method to the indication rather than defaulting to ICSI.

The frozen-transfer advantage has a plausible mechanism but an uneven evidence base. Improved outcomes are attributed to better endometrial receptivity in programmed frozen cycles, the supraphysiological hormonal milieu of fresh stimulated cycles being less favourable to implantation. The meta-analysis supporting this reported a live-birth benefit confined to hyper-responders and to cycles with preimplantation genetic testing, with no difference in normal responders and no gain in cumulative live birth (11). The randomised trials are not uniform. In women with polycystic ovary syndrome, frozen transfer raised live birth after the first transfer from 42.0 to 49.3% (19). In ovulatory women receiving up to two cleavage-stage embryos, there was no difference: 48.7% with frozen and 50.2% with fresh transfer (20). With a single blastocyst, frozen transfer raised singleton live birth from 40 to 50% (21).

Practice has moved faster than the evidence. In the 2019 world report, frozen transfers made up 61.8% of autologous transfers without genetic testing, up from 14.8% in 2015, yet delivery rates per transfer were almost identical for fresh and frozen autologous transfers, at 31.3 and 31.9% (22). The effect here was large: 74.9% versus 51.3%, a difference of 23.6 percentage points. It should be weighed against evidence that frozen transfer carries a higher risk of pregnancy-induced hypertension, pre-eclampsia and placenta accreta (23), and of hypertensive disorders and large-for-gestational-age infants with no clear gain in cumulative live birth (24). There is also a cost dimension specific to this setting: a freeze-all strategy adds cryopreservation and storage charges to a bill already paid out of pocket. A freeze-all strategy might therefore be better offered selectively, to patients with a clear indication, rather than applied by default. The optimal strategy would still depend on patient profile, freezing protocol and laboratory quality.

Oocyte yield was marginally associated with the outcome.

Broader reviews of IVF prognostic factors emphasise age, body mass index, hormonal profile, sperm characteristics and genetic factors (25). The number of oocytes collected has nonetheless been reported as a determinant of success alongside ovarian responsiveness and female age (26). In 400,135 treatment cycles in the United Kingdom, the median yield was nine oocytes, as it was here, and live birth rose with yield up to about 15 (27). Duration of infertility did not reach significance here, though it has been reported as prognostic alongside female age in natural-cycle IVF (28). In linked registry data from 113,873 women, a longer duration of infertility and fewer eggs collected each lowered the chance of live birth (29). Prediction remains imperfect even with far richer data. A model built on 100 prospectively recorded variables in more than 8,000 single-embryo transfers discriminated only moderately (30). The modest pseudo-*R*^2^ reported here is in keeping with that.

One comparison cannot be made here. European registries report clinical pregnancy per aspiration, at about 22% for IVF and 20% for ICSI (31). The same world report gives a delivery rate of 22.9% per aspiration for autologous cycles (22). Our figure is 70.6%, but it is a positive beta-hCG in a woman’s most recent cycle. These are different numerators and different denominators. The two sets of numbers are not comparable and should not be read against each other. A biochemical pregnancy rate will always be the higher of the two, because it counts pregnancies that would have been lost before a scan was ever performed.

### 4.1 Regulatory, ethical and psychosocial context

Infertility is common, and South Asia carries more of it than most regions. In 2010 its prevalence was highest in South Asia, sub-Saharan Africa, North Africa and the Middle East, and Central and Eastern Europe and Central Asia (32). Pooled lifetime prevalence is 17.5% (33), and the age-standardized global burden rose steadily between 1990 and 2017 (34). India alone is estimated to have 27 million infertile couples (35). Treatment has not kept pace. Assisted reproduction in India is concentrated in the private sector, government support is limited, and treatment is neither subsidised nor covered by insurance (36). Across low and middle-income countries, the direct medical cost of a single cycle exceeds average annual income, which places households at risk of catastrophic expenditure (37). The women in this cohort are the ones who could meet that cost.

The burden is not only financial. In a clinic-based study of 300 Indian women with primary infertility, 80% had infertility-specific stress (38). In a second study, low spousal support, financial constraints and social coercion early in marriage predicted distress, and continuous cycles of treatment were described as unaffordable for most patients (39). That infertility causes distress is clear; whether distress lowers the chance of success is not (40). Psychological and physical burden is among the commonest reasons couples give for stopping treatment (41). This bears on the cohort in two ways. Women who reached a second or later cycle had borne the cost and the strain of the first. Women who abandoned treatment before completing a cycle are not in the data.

The regulatory setting changed shortly before this study began. Before the Act, the principal national framework for assisted reproduction in India was a set of guidelines issued by the Indian Council of Medical Research and the National Academy of Medical Sciences in 2005 (42). The Assisted Reproductive Technology (Regulation) Act, 2021 placed clinics and gamete banks under statutory registration and supervision from January 2022 (43, 44). Every cycle in this cohort was performed under it. The Act permits treatment for women aged 21 to 50, the same range used for eligibility here. It also restricts oocyte donation. A donor must be a married woman aged 23 to 35 with at least one living child of her own aged 3 years or more; she may donate only once, no more than seven oocytes may be retrieved from her, and the commissioning party must insure her (43).

These provisions respond to a specific concern. Before regulation, the commercial market in donated oocytes and surrogacy across low-resource Asian countries raised the prospect of exploiting marginalised women (45), and the use of minor girls from poor households as oocyte donors in India has been raised as an ethical problem (46). In the first interview study of Indian egg donors, 19 of 25 gave financial need as their reason for donating (47). The Act’s age floor, lifetime limit and insurance requirement are the statutory answer. This study cannot say how that answer worked in practice. Its records describe recipients, not donors, and hold no information on donor age, circumstances or recruitment. Oocyte donation also moves the determinants of success from the patient to the donor. In national registry data, the disadvantage of a woman’s own oocytes against donated ones widened with her age (17), which is why donor cycles were analysed separately here. They achieved a positive test in 54.5%. In a series of donor cycles at a public tertiary centre in New Delhi, with donors of mean age 25 years, clinical pregnancy was 60% per started cycle (48). The outcomes differ, and the two figures should not be read against each other.

Surrogacy followed a parallel course. Reproductive tourism made India a destination for international commissioning parents, in an industry with an estimated turnover of US$500 million (49). Indian surrogates interviewed before the law changed described surrogacy mainly as a route to improving their family’s economic position, undertaken in the face of social condemnation (50). The Surrogacy (Regulation) Act, 2021 prohibited commercial surrogacy and permits only altruistic arrangements (49, 51). The records used here do not identify surrogacy arrangements or patients travelling from abroad, so neither can be examined. Both nonetheless shaped the market in which a private centre such as this one operates. Regulation has addressed the supply side of assisted reproduction: who may donate, who may carry a pregnancy, and which clinics may operate. It has not addressed the demand side. The cost of a cycle still falls on the household (36, 37). The Act governs how treatment is delivered. It does not govern who can afford it.

### 4.2 Limitations

The outcome is the principal limitation. A positive beta-hCG overestimates clinical pregnancy, and the size of that overestimate in this cohort is unknown. Clinical pregnancy, ongoing pregnancy, miscarriage and live birth could not be ascertained because ultrasound is performed outside the centre and is not captured in its records. What is reported here is an early implantation signal. It is not a clinical pregnancy, and it is not a live birth. The design is retrospective and single-centre, in a private clinic serving a self-paying population. Findings may not generalise to public-sector or lower-resource settings. Residual confounding by unrecorded clinical factors, including sperm parameters, embryo grade and endometrial thickness, is likely.

The cohort is socioeconomically narrow. Seventy-two percent were in the upper or upper-middle strata, against 11.8% in the lower stratum. There is limited variation from which to detect a socioeconomic gradient, and the absence of one here is weak evidence. Within this advantaged cohort, demographic factors did not predict a positive test; that does not rule out demographic effects among lower-income populations, who are barely represented. Treatment expenditure was not recorded. In a wholly self-paying setting, ability to pay may be the more relevant measure of economic position than a composite social-class score, and it could not be examined here.

Selection operates before entry to the cohort. Women who reached a private fertility centre, could pay for a cycle and completed it are systematically different from those who did not. The dataset holds no information on access to care, affordability, barriers to initiating treatment, or discontinuation, so the study cannot speak to inequities arising before a woman enters the fertility-care pathway. What it describes is the performance of one well-resourced private centre serving an affluent population. It is not a success rate for IVF in India, and the findings directly supported by these data should be distinguished from the broader policy implications, which would require evidence on access, affordability and barriers at population level. Age deserves particular caution. Autologous cycles spanned 22–42 years, with only 35 women aged 36 or above and none above 42. Every woman older than 42 received donor oocytes. The study is underpowered to detect age effects and should not be read as evidence that IVF success is independent of age. That it is not independent of age is among the best-established findings in the field.

The unit of analysis is the woman’s most recent cycle, which is not a per-cycle success rate and is not a cumulative rate across cycles. It is also likely to run high. Couples tend to stop after a positive test and to continue after a negative one, and many stop before conceiving (41), so a woman’s latest recorded cycle is more likely than an average cycle to have been successful. Per-cycle and cumulative rates can differ widely, and cumulative estimates depend heavily on what is assumed about those who stop (29, 52). Cycle number itself was not associated with the outcome, and adjusting for it left the treatment effects unchanged. Univariate *p*-values are unadjusted for multiple comparisons.

### 4.3 Conclusion

In this northern Indian fertility centre, a positive pregnancy test after autologous IVF was predicted by treatment decisions and not by patient demographics. That finding holds within a cohort that had already reached a private fertility clinic and could pay for the cycle. Seventy-two percent of these women were in the upper or upper-middle socioeconomic strata. In India, almost all IVF is delivered privately and paid for out of pocket, and a cycle costs more than many households earn in a year. Social position did not appear to separate these women from one another. It might well have separated them from the far larger number of women who never reached a clinic at all. That is the inequity these data cannot measure, and it is probably the larger one.

## Data Availability

The original contributions presented in the study are included in the article/Supplementary material, further inquiries can be directed to the corresponding authors.
